# Design and Fabrication of Low-cost Microfluidic Channel for Biomedical Application

**DOI:** 10.1038/s41598-020-65995-x

**Published:** 2020-06-08

**Authors:** Shailendra Kumar Tiwari, Somashekara Bhat, Krishna K. Mahato

**Affiliations:** 1 Department of Electronics and Communication Engineering, Manipal Institute of Technology, Manipal Academy of Higher Education, Manipal, Karnataka 576104 India; 2 Department of Biophysics, Manipal School of Life Sciences, Manipal Academy of Higher Education, Manipal, Karnataka 576104 India

**Keywords:** DNA, Biomedical engineering, Mechanical engineering

## Abstract

This paper presents the design, simulation and low-cost fabrication of microfluidic channel for biomedical application. Channel is fabricated using soft lithography technique. Printed Circuit Board (PCB) is used to make the master for the channel. Channel pattern is transferred on PCB plate using toner transfer technique followed by ferric chloride etching. Paper also discusses, the issues involved in PCB based master fabrication and their viable solutions. Glass is used as substrate material and the channel is made of Sylgard 184 Polydimethylsiloxane (PDMS). Channel is interfaced with a syringe pump to observe the fluid flow. To predict the behavior of the channel, FEM simulation is performed using COMSOL Multiphysics 5.2a. There is a good match between the theoretical, simulation and test results. Finally, to test the biocompatibility of the channel, genomic DNA is passed through the channel and gel electrophoresis analysis is performed.

## Introduction

Microfluidics deals with the study of devices that can handle the very small amount of liquid down to femtoliter with the help of small channels and reservoirs. The dimensions of microfluidic devices range from ten to hundreds of micrometers^1–5^. These devices have promising applications in biological analysis^6–9^ chemical analysis^10^, optical communication^11^, cooling of Integrated Circuits (ICs)^12^, and many more. In microfluidics, scaling down the device dimensions to microscale reduces the amount of sample and reagents required to perform the assay, resulting in a huge saving in cost and the reduction in the amount of waste produced. Due to low fabrication cost, it can easily be disposed-off. This also reduces the risk of cross-contamination between the tests. The portable analysis devices that can perform the necessary analytical tests outside the central facility at the remote location or in the vicinity of the patient have a huge demand in the biomedical industry. Performing the assay at the location of patient results in real-time test data, which can help medical experts to intervene timely, and this can improve the clinical outcome of the patient^13–17^. Fabrication of low-cost sensors, transducers, and biomedical analysis devices were possible due to advances in Micro Electro Mechanical Systems (MEMS) technology. In this technology there are two popular fabrication approaches, first uses silicon and the second, polymers^18–20^. Polymer-based MEMS devices widely used in the biomedical industry due to advantages it offers such as biocompatibility, ease of fabrication with the minimum facility, low-cost, ease of disposal and a minimal volume of sample and reagents requirement^9,21,22^. These polymer-based devices are also known as BioMEMS or microfluidic devices^23^. The microfluidic analysis chip, which enables the complete biomedical analysis without the external intervention, are called micro Total Analysis Systems (μTAS) and are very useful for the Point-of-Care (POC) applications^15^. Microchannels, microvalves, micropumps, and micromixtures are few popular examples of microfluidic devices.

Due to the large demand for microfluidic devices, several research groups are working towards the design and fabrication of cost-effective biomedical analysis chips. The device is fabricated in two stages, the first master (the reverse pattern of the microfluidic channel) fabrication and the second, master pattern transfer on polymer using soft lithography. The masters made of SU8 photoresist^24–26^, 3-D printing^27^, Soldering wire^28^, and, PCB^29,30^ are popular in the fabrication of microfluidic devices.

Fukuba *et al*.^24^ have reported the master made by patterning a 100 μm thick SU8 photoresist on a silicon substrate for microfluidic PCR chip. The issue involved with this approach is some unwanted tilt after spinning SU-8 and before baking results in wrinkles on the master due to the large thickness of SU-8 (100 μm). Any imperfection on the master is translated on all the patterns made using the master.

Kumar *et al*.^25^ have reported the fabrication of flow-through microchannel with different channel height for PCR application. The master is fabricated using a three-layer lithography process, the first layer of SU-8 acts as an adhesion promoter, the second SU-8 50 layer having a thickness of 50 μm decides the channel height of the transition zone, and the third SU-8 2050 layer with thickness of 100 μm fixes the channel height in reaction region as 150 μm (50 μm+100 μm). The requirement of two masks increases the overall device fabrication cost and misalignment of masks during the UV exposure results in the non-working microfluidic channel. Also, fabrication is only possible under cleanroom facility.

3-D printing is a low-cost approach to master manufacturing^27^, and it requires the following steps: first, 3-D design of the master created with the help of CAD tool. Second, CAD design is converted to Standard Tessellation Language (STL) format, and third, the design is printed with the 3-D printer. The 3-D printed master requires postprocessing to improve the surface smoothness.

Song *et al*.^28^ have reported fabrication of a low cost circular microfluidic channel using metal wire removal process. Soldering wire with a circular cross-section having a diameter 300 μm is used as a master for the microchannel. In this approach, the channel diameter is limited by the diameter of soldering wire and master is having a single-use lifetime. The removal of soldering wire requires vacuum and precise temperature control. Also, traces of solder wire in the microfluidic channel will have an adverse effect on the biological sample.

Printed Circuit Board based masters for microfluidic devices is a cost-effective alternative for master fabrication, and it does not require any cleanroom facility^29,30^ and hence, most suited to the small research groups who do not have access to the cleanroom facility. The thickness of copper on copper clad laminate decides the height of the microfluidic channel.

Table 1 shows the comparison between the various master fabrication approaches. The SU-8 master uses silicon or glass as substrate material due to their compatibility with IC fabrication process, whereas soldering wire-based master requires a base material which helps to shape the soldering wire. The 3-D printed master are commonly made of ABS plastic. During the 3-D printing of master, base also gets printed and hence, it does not require any special substrate material. Printed Circuit Board master uses a copper clad laminate, and hence PCB master also does not require any special base material. Printed Circuit Board based master fabrication is the most economical fabrication option and it does not require any post-processing.

**Table 1:** Comparison of various master fabrication techniques.

	SU 8 master^24–26^	3-D Printed^27^	Soldering wire^28^	PCB^29,30^
Substrate	Si/Glass	ABS	Not Applicable	Copper clad
Number of Masks	01–03	Nil	Nil	1
Photoresist	Negative	Not Applicable	Not Applicable	Not Applicable
Fabrication Time	More	Less	Less	Less
Cost	High	Low	Low	Low
Channel Height	SU-8 thickness	Pattern height	Wire diameter	Copper height
Master Life Time	Multiple use	Multiple use	Single use	Multiple use

**Figure 1:**
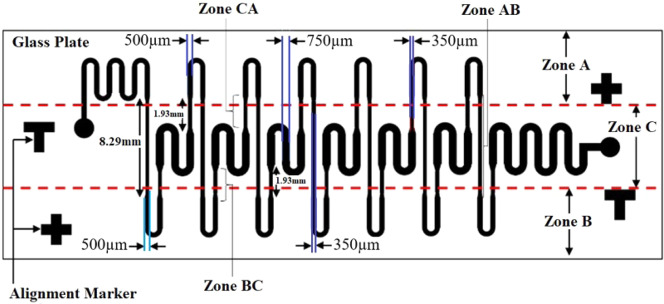
Schematic of the microfluidic channel.

Present work deals with the design, simulation, and fabrication of low-cost microfluidic channel with a rectangular cross-section using PCB based master. The schematic of the microchannel is shown in Fig. 1.

## Governing Equations

If the smallest dimension of a channel ranges from 10 μm to 200 μm, then channel can be categorized as microchannel^1^. The geometry of the microfluidic channel decides the residence time of the fluid. Therefore, dimensions of the different segment of the channel are selected such that, the ratio of time spent by the fluid at Zone-A, Zone-B, & Zone-C is 1: 1: 2 (Fig. 1). The residence time of fluid for each zone is given by^25^,

1 $${t}_{i}=\frac{{l}_{i}{w}_{i}{h}_{i}}{Q}$$

Here, i = A, AB, B, BC, C and CA are the various zones and *l*, *w*, and *h* are length, width, and height of the channel in each zone.

Variable *Q* is the volumetric flow rate given by,

2 $$Q={A}_{i}{V}_{avg}$$

Here, *A*_*i*_ represents the cross-sectional area of the microfluidic channel, and *V*_*avg*_ is the average velocity of the fluid.

The head loss must be calculated for the device operation and the maximum allowable head loss for PDMS bonding to remain intact should be within 200*kPa*. The head loss expression is given as,

3 $${h}_{Li}\,=\,f\frac{{l}_{i}}{{D}_{hi}}{\rho }_{f}\frac{{{V}_{avg}}^{2}}{2}$$

Where, *f* is Darcy friction factor, *D*_*hi*_ is the hydraulic diameter, and *ρ*_*f*_ is the fluid density.

The Darcy friction factor for laminar flow in microchannels can be approximated as,

4 $$f=\frac{{\beta }_{i}}{R{e}_{Dhi}}$$

In Eq. (4), *β*_*i*_ is a constant dependent on the geometry of the channel and is given by^1^,

5 $${\beta }_{i}=24(1-1.3553{\alpha }_{i}+1.9467{\alpha }_{i}^{2}-1.7012{\alpha }_{i}^{3}+0.9564{\alpha }_{i}^{4}-0.2537{\alpha }_{i}^{5})$$

Here *α*_*i*_ represents the ratio of height to width of the channel and for the present case it is less than 1. *Re*_*Dhi*_ is the Reynolds number which depends on the hydraulic diameter.

In present work, *β*_*i*_ is 16.97 for Zone A and Zone B, 15.63 for transition Zone i.e. Zone AB, Zone BC and Zone CA and 18.56 for Zone C. The hydraulic diameter for different zones *D*_*hi*_ for the rectangular channel is given as,

6 $${D}_{hi}=\frac{4{A}_{i}}{{P}_{i}}$$

With *A*_*i*_ = *w*_*i*_*h*_*i*_ and *P*_*i*_ = 2(*w*_*i*_ + *h*_*i*_) Eq. (6) reduces to,

7 $${D}_{hi}=\frac{4{w}_{i}{h}_{i}}{2({w}_{i}+{h}_{i})}$$

Reynolds number, which depends on the hydraulic diameter (*D*_*hi*_) is given as,

8 $$R{e}_{Dhi}=\frac{{\rho }_{f}{V}_{avg}{D}_{hi}}{\mu }$$

Here μ represents the dynamic viscosity of the fluid.

The channel dimension at each zone of the microfluidic channel is given in Table 2. To have time spent in Zone C to be double that of A and B, the width of Zone C is kept at 750 μ*m* whereas, Zone A and Zone B are only 500 μ*m* wide. It is also observed from Table 2 that minimum width (350 μ*m*) of transition zone i.e. Zone AB, Zone BC, and Zone CA will result in minimum residence time (Eq. (1)).

**Table 2:** Specifications of microchannel used for simulation.

Zone	Length(mm)	Width (μ*m*)	Height (μ*m*)
A	5.68	500	172
AB	8.29	350	172
B	5.68	500	172
BC	1.93	350	172
C	13.0	750	172
CA	1.93	350	172

## Fabrication

The fabrication process of reported microchannel can be divided into two parts: first, master fabrication, and second, microchannel fabrication using soft lithography. The items required for the master fabrication are: photo paper, laser printer, paper cutter, iron box, window glass cleaner, PCB plate, hacksaw blade, 98% anhydrous ferric chloride, kitchen scrubs (steel wool preferred), and acetone.

**Figure 2:**
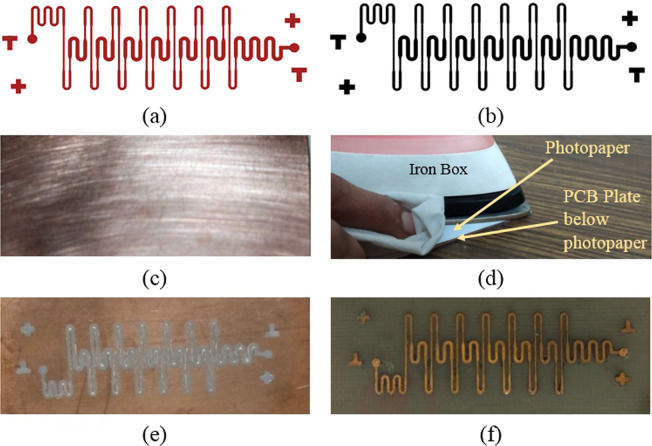
PCB based master fabrication steps (**a**) Layout (**b**) Photopaper print (**c**) PCB plate (**d**) Toner transfer (**e**) Channel pattern on PCB (f) Master.

The channel layout is designed using L-Edit (Tanner EDA V14.13 Windows) software (Fig. 2(a)) and a high-resolution laser print of the layout is taken using the 1200dpi HP-laser printer, on glossy side of the photo paper (Fig. 2(b)). To reduce the etching time, the PCB board is cut to required size and if PCB is having double-sided copper layer, one side can be sealed with tape. The copper plate is scrubbed to remove any dust with scrubber followed by acetone cleaning (Fig. 2(c)). The printed design from photopaper is cut into the size of PCB plate and window glass cleaner solution is sprayed on it. After 15 *s*, the cleaner solution coated paper is placed on the PCB plate in such a way that printed side is placed facing the copper layer on PCB plate. With the help of iron box set to its highest temperature, pressure is applied on photo paper for 90 *s* by placing it over PCB plate (Fig. 2(d)). The paper is removed gently from the PCB plate with the help of lukewarm water. At the end of this process, the pattern is transferred from photo paper to the PCB plate (Fig. 2(e)). Finally, the patterned copper layer is etched with the help of ferric chloride solution. After development, master is cleaned with acetone to remove any left-out tonner. Figure 2(f) shows the master which is ready to be used for the fabrication of microchannel.

**Figure 3:**
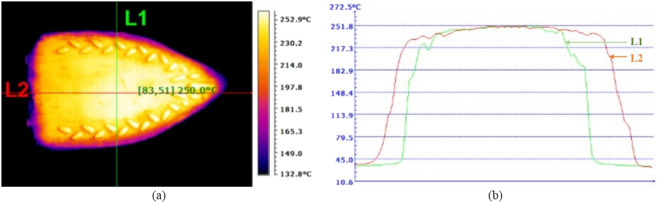
(**a**) Thermal image of iron box (**b**)Temperature profile of iron box along the line L1 and L2.

Some of the issues involved in the master fabrication using PCB plate and possible solutions are presented next. The low or medium operating temperature of iron box results in poor pattern transfer. Therefore, it must be insured the iron box set to its highest temperature. From thermal image shown in Fig. 3(a) it can be seen that the iron box operating at 250 ^ο^C. Figure 3(b) shows the temperature profile along the line L1 and L2, it can be seen that it has uniform temperature across the plate. Further, Copper is a good thermal conductor while pattern transfer it may bring entire copper plate to almost a uniform temperature. Hence, slight variation in temperature of iron box will not be an issue for the master fabrication.

**Figure 4:**
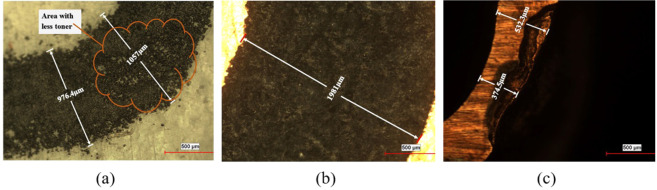
Defect in master due to poor print quality and masking layer (**a**) Patches on print (**b**) Transferred toner layer on PCB plate (**c**) Defect in master due to poor toner masking layer.

The height of the microfluidic channel is limited by the thickness of the copper on the PCB. In the present work, PCB with a copper layer thickness of 100 μ*m* was used but when the thickness of the copper layer was measured with screw gauge, it was varying from 172 μ*m* to 192 μ*m* at various regions of PCB plate. Thus, it is important that, the quoted numbers are not used as it is in calculation and simulation, instead actual measured values are used. Also, as much as possible, it is important to use PCB plate with uniform copper layer thickness. In the market copper clad laminates with 18 μ*m*, 35 μ*m*, 70 μ*m*, 100 μ*m*, and 285 μ*m* thickness are available. As per the requirement of the channel height, appropriate PCB plate can be selected. A 1200dpi print is good enough for good uniformity and prints with resolution lower than this will lead to patches in the pattern transferred on a copper plate and results in the defect in the master. Figure 4(a) illustrates one such defect in the form of patches on the printed layer. Figure 4(b) shows the transferred toner layer from photo paper to PCB plate. Figure 4(c) shows a part of the master after development, with a defect on the master. To overcome the problem of poor toner transfer shown in Fig. 4, after transferring the patterns on the copper plate, these defective patches can be covered with the help of fine tip permanent marker.

If the copper layer thickness on PCB plate is large, then it must undergo long etch time. Therefore, it is suggested that after a fixed interval of time, the masking layer on PCB plate is repaired using permanent marker.

**Figure 5:**
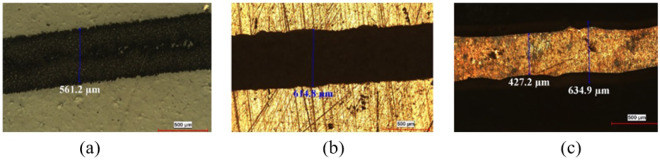
Feature size variation from photopaper to master (**a**) Toner layer on photopaper (**b**) Toner layer on PCB plate (**c**) Master after development.

From Fig. 5(a) it can be observed that, the line width of toner on paper is 561.2 μ*m*, whereas, as seen in Fig. 5(b), the line width of the toner layer on the copper plate is 614.8 μ*m*. Thus, there is spread in the toner line width during pattern transfer process. The increase in line width can be controlled in many ways. As a first approach, the pressure applied can be optimized to obtain the optimum line width. Another solution is to reduce the quantity of window glass cleaner spray to achieve tight control on line width spreading. As a third solution, the line width in layout can be reduced to accommodate this increase in the line width after pattern transfer.

Figure 5(c) shows the line width of the copper track after etching the PCB plate. It is observed that the line width has been reduced due to sidewall etching. By standardizing the etch time and by controlling the side wall etching the problem can be reduced to a certain extent.

**Figure 6:**
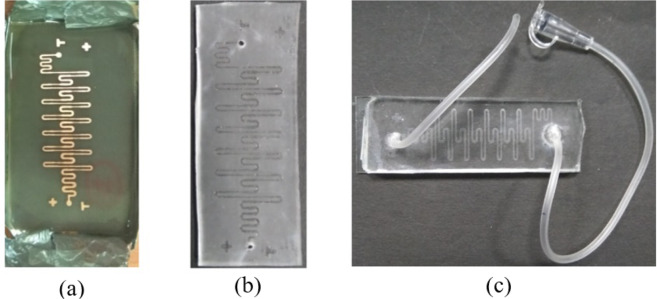
Various stages of channel fabrication (**a**) Cured PDMS on master (**b**) Channel pattern transferred on PDMS (**c**) Final channel with inlet and outlet pipes attached.

For the microfluidic channel fabrication, soft-lithography technique is used. Following items are required for fabrication: master, acetone, Sylgard 184 elastomer kit (PDMS), vacuum pump, desiccator, ethanol, DI water, hot plate, aluminum foil, glass slides, spin coater and flexible tube. The fabrication process starts with the cleaning of master with acetone. A mixture of PDMS and curing agent in the ratio of 10:1 is poured on the master and kept in the vacuum to remove air bubbles. After degassing, it is cured at 95°C for 20 minutes (Fig. 6(a)). Cured PDMS is carefully peeled-off and inlet and outlet holes are created on this PDMS layer with the help of a 2 *mm* diameter biopsy punch. The resulting channel structure is as seen in Fig. 6(b). The cured PDMS is cleaned with ethanol followed by DI water and dried. Finally, the microfluidic structure is bonded to the glass substrate and tubing is done to interface the channel with syringe pump. The resulting device is shown in Fig. 6(c).

**Figure 7:**
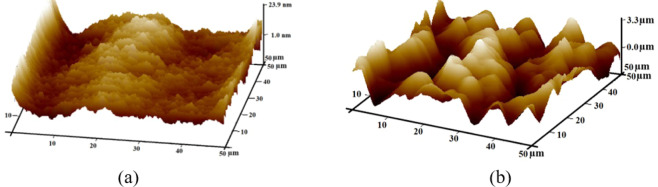
Surface morphology of (**a**) Copper region (**b**) Copper etched region on a PCB plate captured using AFM.

The surface morphology of copper lines remaining after etching unwanted copper is captured using AFM (Fig. 7(a)) and it shows a maximum surface roughness of about 6.08 *nm*. As these copper lines constitute the channel region in PDMS, the channels formed will thus have smooth surface. However, the AFM study of etched regions (Fig. 7(b)) shows a surface irregularity of the order of 3.3 μ*m*. This profile gets duplicated on the cured PDMS layer.

**Figure 8:**
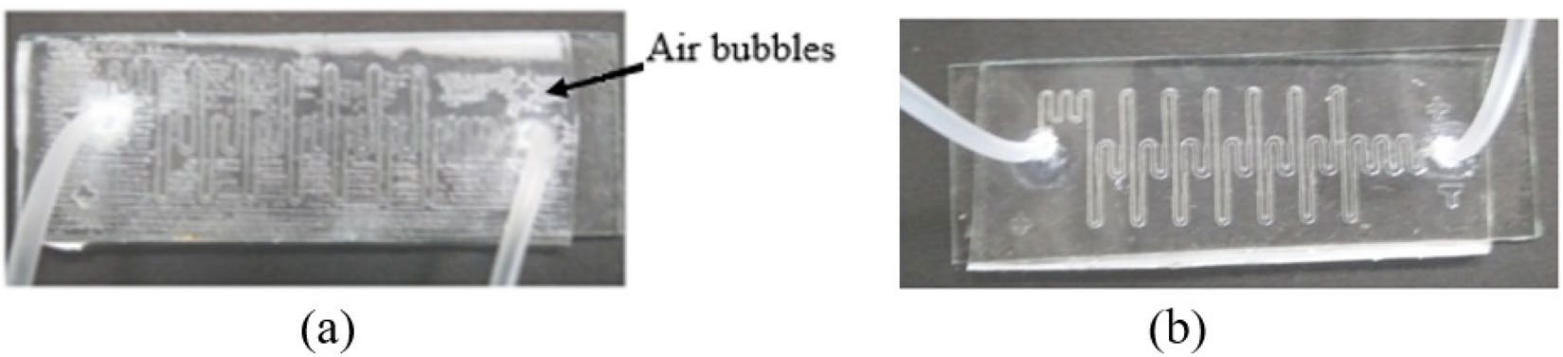
Image of glass-PDMS bonded layer (**a**) With air bubbles (**b**) Without air bubbles.

While bonding, these uneven surfaces result in the air pockets between the glass plate and the PDMS layer as shown in Fig. 8(a). After placing the PDMS coated glass and PDMS channel in contact, the entire structure was placed in vacuum for 15 minutes followed by the application of gentle force with hand to eliminate all the trapped air bubbles. The image of the bonded layer is shown in Fig. 8(b), and it can be observed that the bonded structure is now devoid of air bubbles.

**Figure 9:**
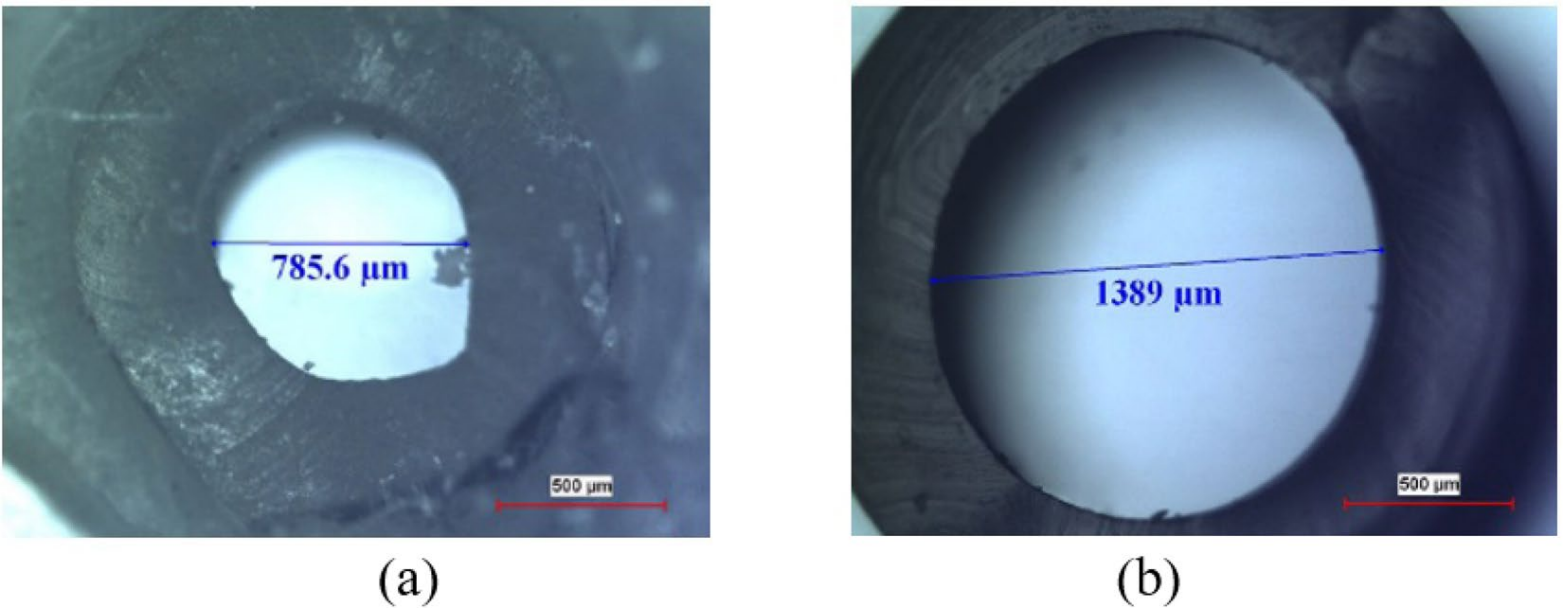
(**a**) Inlet diameter after tubing (**b**) Diameter of the rubber tube used for channel interface.

If the diameter of the biopsy punch and rubber tube used for interfacing the channel with external world, are very close to each other, it is possible that during the interconnection, tubing may get compressed at the inlet and the outlet of channel, one such situation is shown in Fig. 9(a). Even though, diameter of tubing is 1500 μ*m*, it is compressed to 785.6 μ*m* after interconnection. Due to deformation in the small piece of rubber tube the measured diameter shown in Fig. 9(b) is 1389 μ*m*. Therefore, compression of tubing at the channel interface will affect the normal flow in the channel.

**Figure 10:**
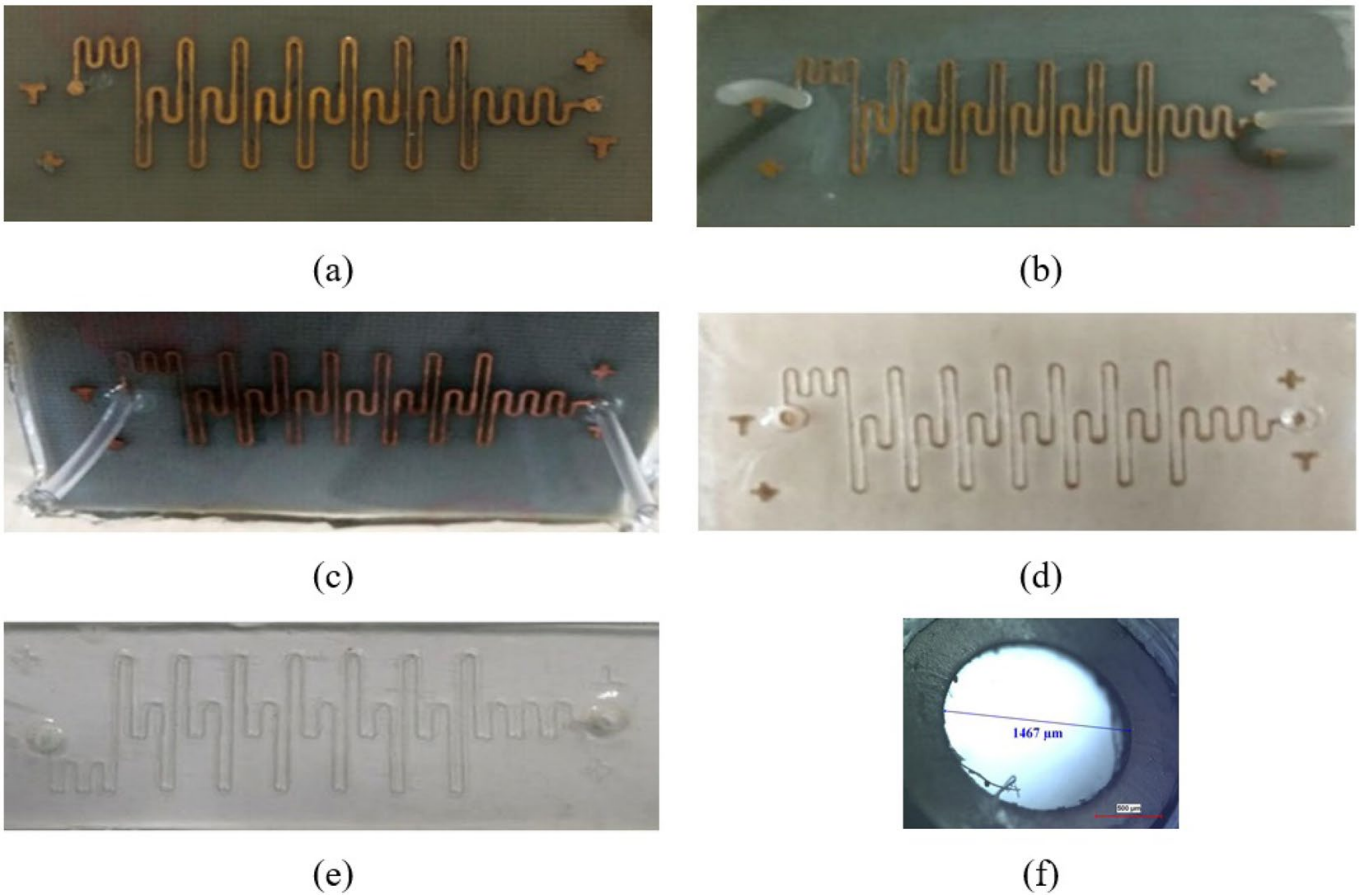
Microchannel fabrication steps (**a**) Master (**b**) Master with tube glued on it (**c**) Cured PDMS mixture on master (**d**) Peeled PDMS layer (**e**) Channel after PDMS layer and glass bonding (**f**) Optical microscope image of inlet point of channel.

Following steps are adopted to overcome this problem, the PCB based master is cleaned with ethanol followed by heating at 40 °C for 3 minutes (Fig. 10(a)). Small piece of rubber tubes are glued directly at the inlet and outlet points of the master (Fig. 10(b)). The mixture of PDMS and bonder in the 10:1 proportion is poured on the master and kept for degassing till all the trapped air bubbles are removed followed by curing at 95 °C for 20 minutes. The cured PDMS on master is shown in Fig. 10(c). The cured PDMS is peeled off carefully, and the resulting channel pattern is shown in Fig. 10(d), As seen, the PDMS layer has the prefabricated inlet and outlet holes for the required tubing size. The PDMS layer with channel pattern is bonded with the glass slide and the bonded structure is shown in Fig. 10(e). The zoomed view of the inlet hole is shown in Fig. 10(f); it can be seen that inlet hole is having a perfect circular shape and its diameter is 1467 μ*m*. This approach also obsoletes the requirement of biopsy punch. Further, if the application requires an inclined inlet and outlet, the rubber tubes can be cut at a required angle and glued on the master.

## Simulation

**Figure 11:**
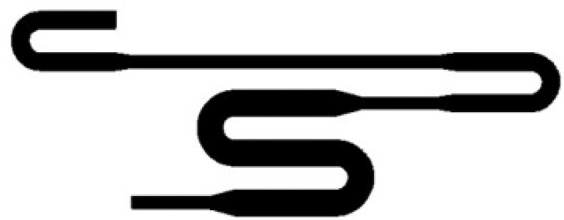
Mesh model of the microchannel.

The specifications shown in Fig. 1 and Table 2 are used for modeling the microfluidic channel using the COMSOL Multiphysics simulation tool. Laminar flow and the particle tracing for fluid flow physics are used for the channel simulation. The physics-controlled extremely fine mesh is used for the simulation. Even though there are 7-cycles, to save the computation time, the simulation is performed for a single cycle. The mesh model used for the study is shown in Fig. 11. Stationary and time-dependent simulation is carried out to obtain the fluid velocity profile and the residence time in a microfluidic channel respectively. The inlet fluid velocity is set to 0.00246 *m/s*. The residence time simulation is carried out from 0–12 *s* in steps of 0.01 *s*. Water is used as the fluid for the simulation and all the material properties for the simulation is taken from the COMSOL material library.

## Results And Discussion

**Figure 12:**
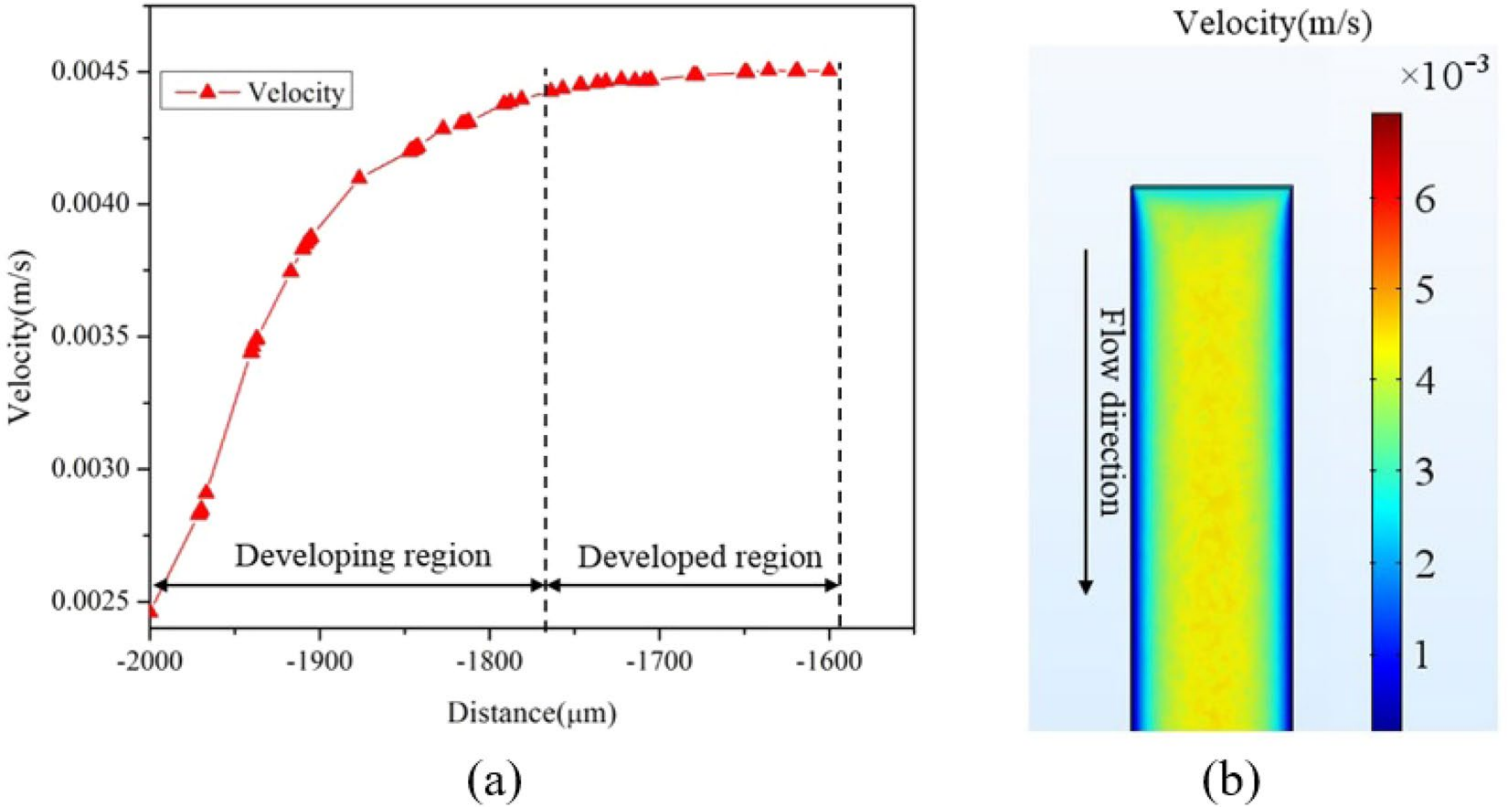
Velocity profile in Zone A (**a**) Along the length (**b**) Along the surface.

The 1 cycle of microchannel is divided into Zone A, AB, B, BC, C, and CA (Fig. 1). Fluid enters the channel from Zone A and leaves from Zone CA. As fluid enters the channel, it requires some distance known as entrance length to be travelled before it attains the maximum velocity. Velocity profile along the length and the surface in Zone A is shown in Fig. 12(a) and (b) respectively. The length of the developing region for laminar flow is given by *L*_*E*_ = *D*_*hi*_*Re*_*Dhi*_. This results in an analytical value of 223 μ*m* for entrance length in Zone A. As seen in Fig. 12(a), entrance length obtained using simulation for Zone A is 230 μ*m*, which has a good agreement with the analytical value.

**Figure 13:**
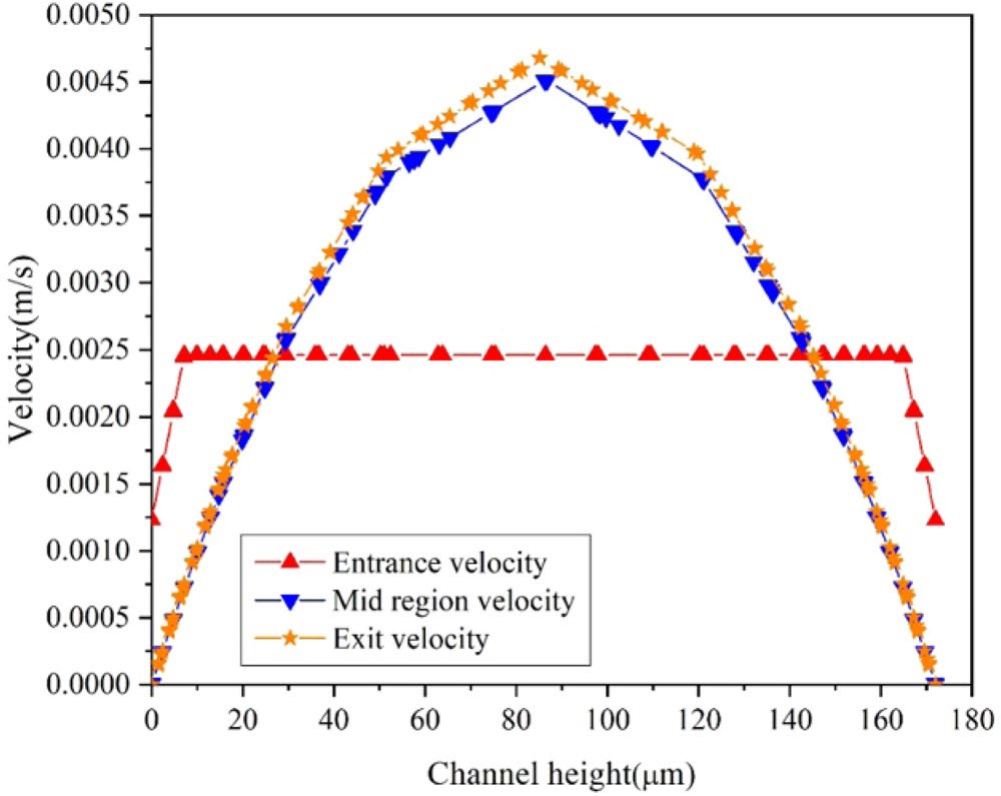
Velocity profile of Zone A along the vertical cut line passing through the middle of the channel.

The velocity profile along the vertical cut line passing through the middle of the channel is shown in Fig. 13. At the entrance of the channel, input velocity is set to 0.00246*m*/*s* and it is constant throughout the inlet of the channel. The fully developed velocity profile is presented as mid region velocity plot in Fig. 13. As flow progresses in the channel, it attains a peak velocity of 4.5 × 10^−3^*m*/*s*. It is also seen in Fig. 13 that the exit velocity (4.55 × 10^−3^*m*/*s*) is slightly more than the fully developed velocity, which may be due to the decrease in the cross-sectional area of the channel after the exit point of Zone A.

**Figure 14:**
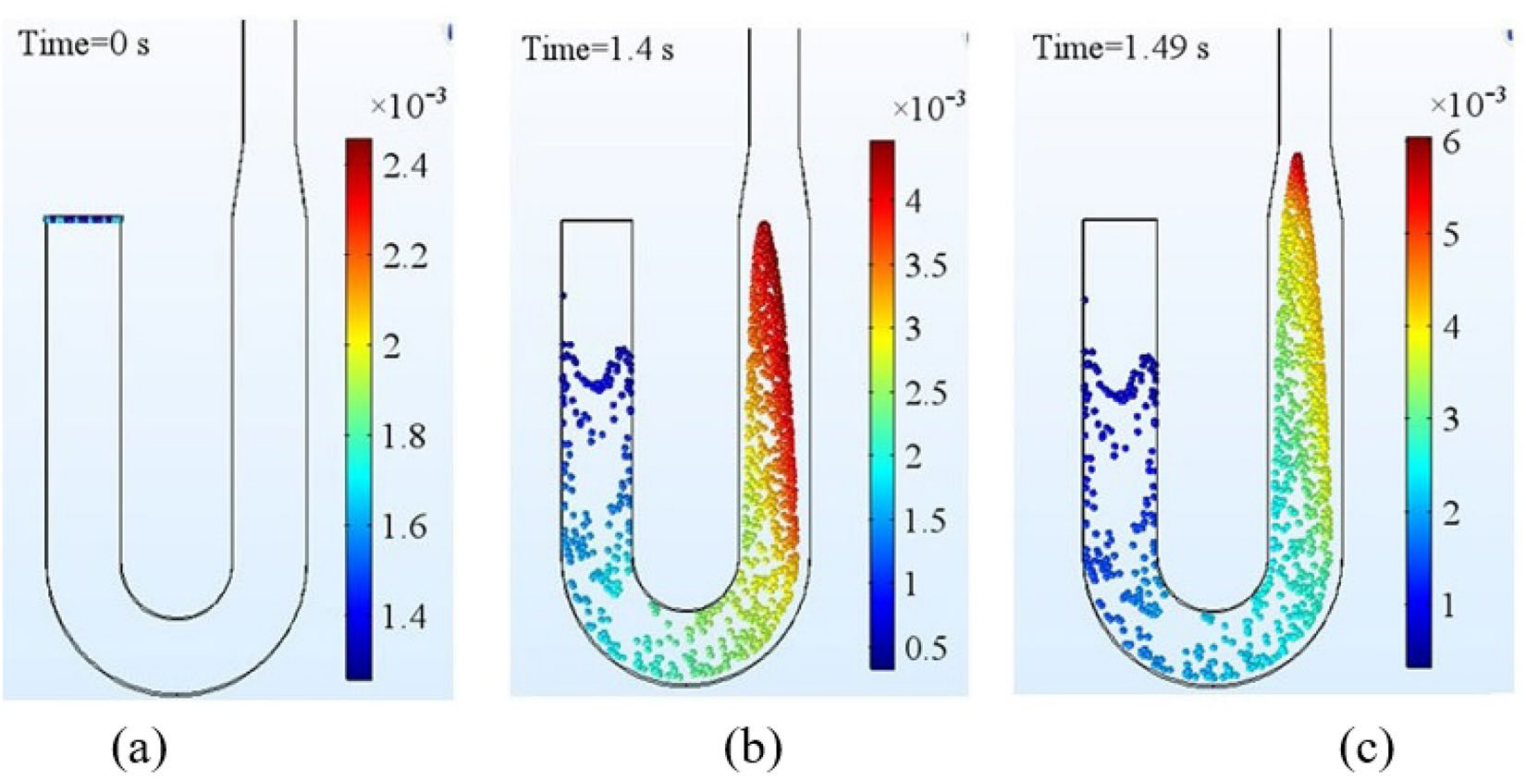
Particle tracing results for Zone A at (**a**) 0 (**b**) 1.4 (**c**) 1.49 seconds.

The outcome of particle tracing simulation for Zone A is shown in Fig. 14. The flow begins in the channel at 0 *s* (Fig. 14(a)) and the first particle reaches the exit of the channel at 1.4 *s* (Fig. 14(b)) resulting in a residence time of 1.4 *s*. The analytical residence time for Zone A is 1.43 *s*. The difference in the analytical and simulation result can be due to not considering the bending effect of channel in the analytical calculation. Also, to keep the analytical model simple, the converging and diverging portion of the channel is neglected. From Fig. 14(b) and (c), it can be observed that, it takes around 0.09 *s* to cross the converging section of the channel immediately after the Zone A.

If fluid moves from larger to smaller cross-section, segment velocity increases and vice versa. From the continuity equation, we know that,

9 $${A}_{1}{V}_{1}={A}_{2}{V}_{2}$$

Where *A*_1_ and *V*_1_ are the cross-sectional area and velocity of the first region and *A*_2_ and *V*_2_ are the cross-sectional area and velocity of the second region.

**Figure 15:**
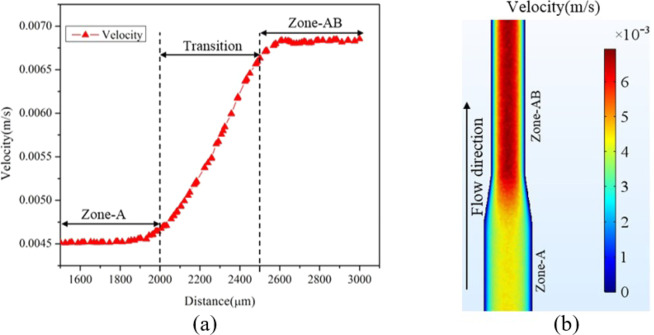
Velocity variation from Zone A to Zone AB (**a**) Along the length (**b**) Along the surface.

The height of the microchannel is 172 μ*m* and width of each zone can be found from Fig. 1. Using these two, the cross-sectional area of each zone can be calculated. Using the peak velocity of Zone A taken as 4.51 × 10^−3^*m*/*s* in Eq. (9), the peak velocity of Zone AB is found to be 6.44 × 10^−3^*m*/*s*. The peak velocity of Zone AB obtained by simulation is 6.71 × 10^−3^*m*/*s* (Fig. 15(a)). The initial variation in the velocity at the beginning of Zone AB is due to a developing flow. The variation in simulation and the analytical result is about 4.0%. The surface velocity profile for channel segment from Zone A to Zone AB is shown in Fig. 15(b). As the channel width decreases the velocity of fluid increases.

The peak velocity at various points of the channel is listed in Table 3. It is observed for Zone B and Zone C that mid-region velocity is slightly less than the input velocity. This is because of the tapering of the channel in the inlet and outlet region of the respective zones.

**Table 3:** Peak velocity of fluid in various zones of the channel.

Zone	Peak Velocity (10^−3^*m*/*s*)		
	Inlet	Mid-region	Exit
A	2.46	4.50	4.55
AB	6.63	6.82	6.56
B	4.68	4.50	4.68
BC	6.64	6.84	6.56
C	3.01	2.81	2.99
CA	6.51	6.84	7.06

**Figure 16:**
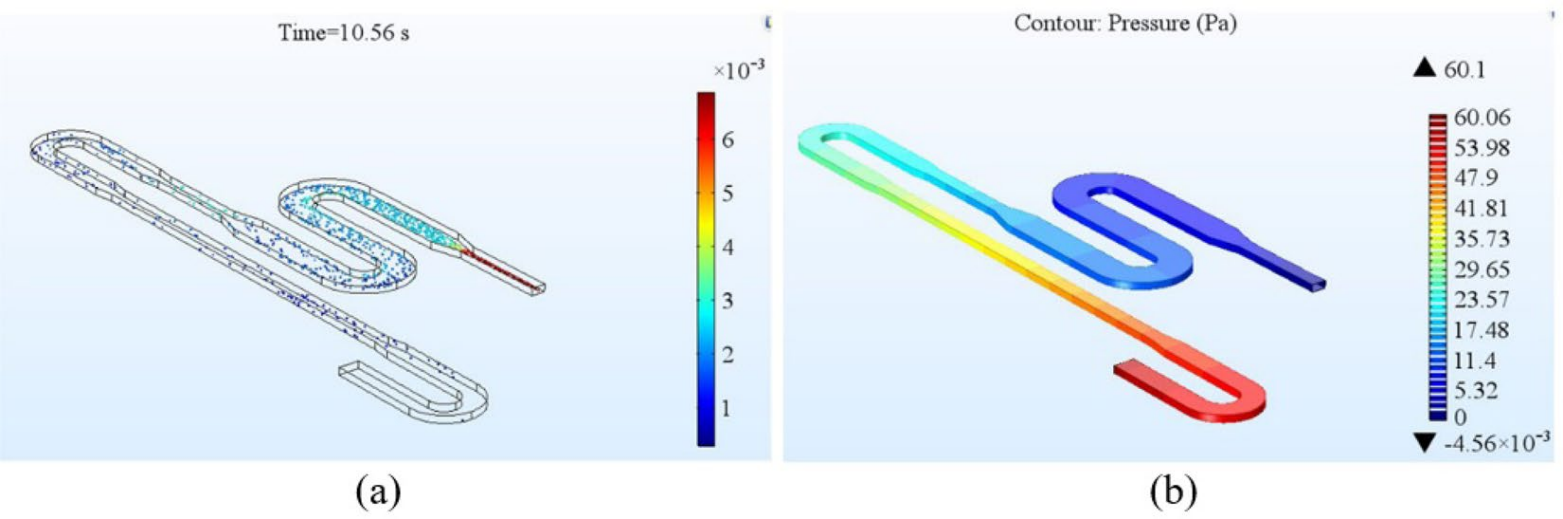
Simulation results for 1 cycle of the channel (**a**) Residence time (**b**) Pressure drop along the channel length.

The simulation result obtained for residence time of the single cycle channel is shown in Fig. 16(a). The maximum allowable pressure for the PDMS - glass bonding to be intact is 200*kPa* and pressure drop along one cycle is 60 *Pa* (Fig. 16(b)), this results in a total pressure drop of 1980*Pa* for a 33 cycle PCR set-up, which is much below the threshold value.

Table 4 shows the residence time obtained analytically and by simulation for various segment of channel. It can be seen from the Table 4 that maximum difference is less than 6%.

**Table 4:** Residence time of fluid at various zones of the channel.

Zone	Residence Time(s)		% Difference
	Theoretical	Simulation	
A	1.43	1.40	2.0
AB	1.46	1.43	2.0
B	1.43	1.47	2.7
BC	0.34	0.36	5.8
C	4.93	4.93	0.0
CA	0.34	0.36	5.8

**Figure 17:**
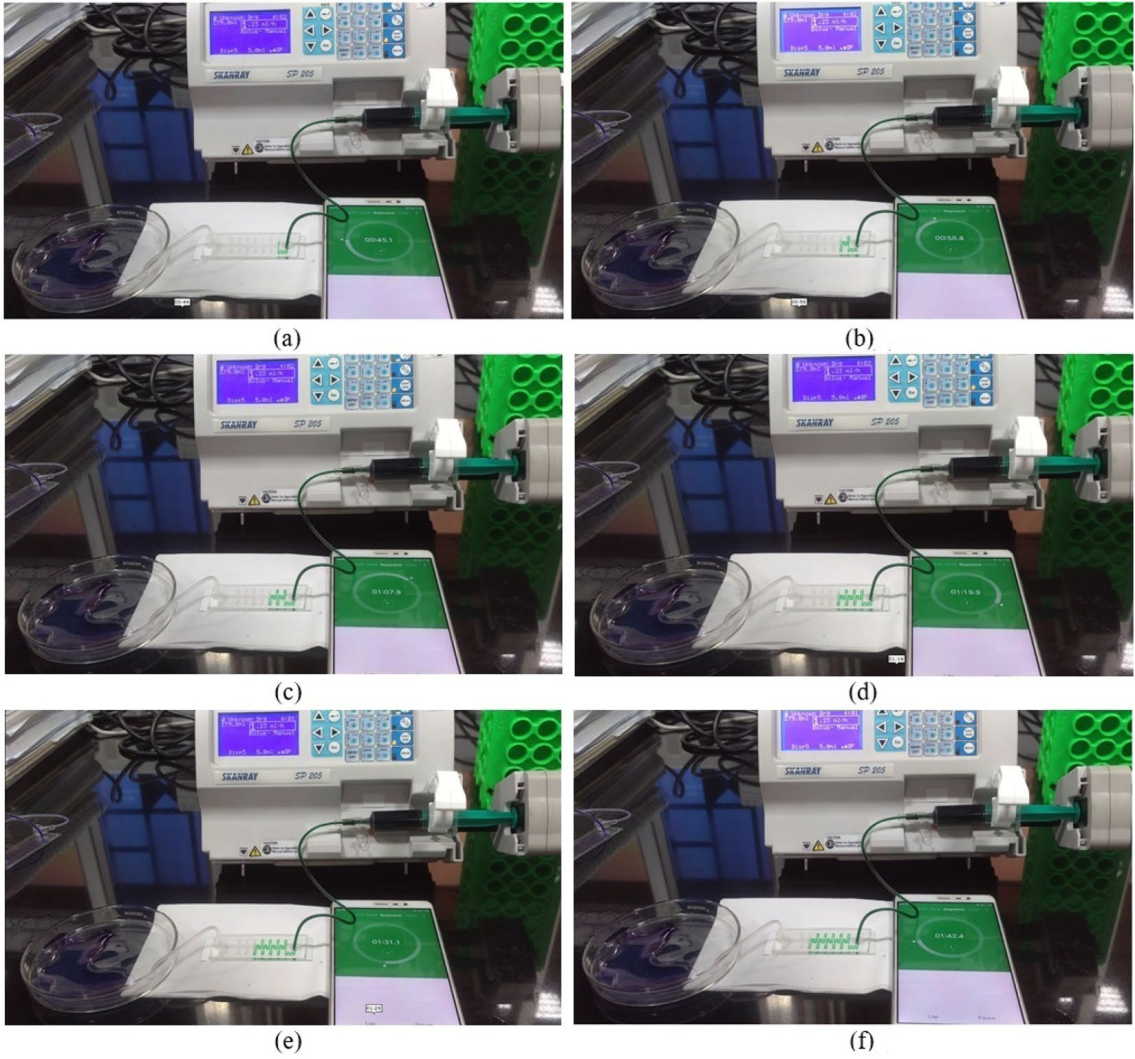
Channel test setup for a residence time.

Test setup for the microfluidic channel is shown in Fig. 17. It requires a syringe pump (SKANRAY SP-205) to control the flow rate, a 5 *ml* syringe (DISPOVAN), Channel Under Test (CUT), a Petri dish to collect the fluid from the outlet of CUT and a timer. The flow rate of the syringe pump is set to 1.23 *ml/hr*, leading to a volumetric flow rate of 3.42 × 10^−10^*m*^3^/*s*. To avoid any kind of discrepancy in the measurement of residence time, initial few turns of channel are not considered in measurement because due to bending it may modify the input velocity. From Fig. 17(a), it can be observed that flow begins at 45.1 *s* and completes 1 cycle at 56.5 *s* (Fig. 17(b)). The difference in two timings gives the residence time of 11.4 *s*. Similarly, the second, third, fourth, and fifth cycles complete at 67.9 *s* (Fig. 17(c)), 79.9 *s* (Fig. 17(d)), 91.1 *s* (Fig. 17(e)), and 102.4 *s* (Fig. 17(f)) and resulting residence times are 11.4 *s*, 12.0 *s*, 11.2 *s* and 11.3 *s* respectively. Therefore, the average residence time per cycle is 11.46 *s*. When compared to residence time of 10.56 *s*, obtained using simulation, the measured residence time of 11.46 *s* differs by 7.8%. The discrepancy in the simulated and measured residence time values are reasoned-out in the next paragraph.

When compared to the expected channel dimensions (Fig. 1), the actual dimensions differ by as much as 45 μ*m* (instead of 500 μ*m*, Zone B has a width of 545 μ*m* (Table 5)). Also the thickness of copper layer on master varies from 172 μ*m* to 192 μ*m*, which leads to the average thickness of 182 μ*m* (Table 5). These differences could be one of the reasons for the 7.8% variation in the two results.

**Table 5:** Channel dimension of various zones acquired from master.

Zone	Length (mm)	Width (μ*m*)	Height (μ*m*)
A	5.68	545	182
AB	8.29	390	182
B	5.68	545	182
BC	1.93	390	182
C	13.0	728	182
CA	1.93	390	182

**Figure 18:**
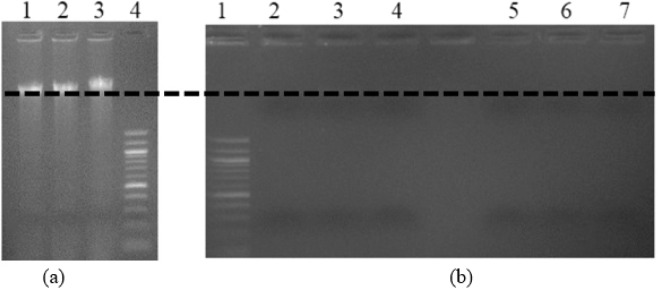
Gel image (1%) representing the (**a**) DNA after running through the channel (**b**) Trapped DNA being flushed-out after each wash.

To demonstrate that the channel material does not cross-react with DNA, channel surface does not damage DNA, and DNAs are not trapped inside the channel, 33 μ*L* of genomic DNA was run twice in the channel. Gel electrophoresis (Fig. 18) is carried-out using the given DNA sample and the DNA sample which has passed through the channel. The first column in Fig. 18(a) shows the given DNA sample before passing through the channel. The second and third columns in Fig. 18(a) show the DNA samples that have passed through the channel and the fourth column is the marker. The 100 bp DNA ladder marker is used in the Agarose gel analysis. However, the objective of the experiment to find out the damage caused to DNA while passing through the channel and retention of the DNA in the channel. Therefore, marker ladder is not labelled. The band formation at the same level in columns 1 to 3 in Fig. 18(a), indicates that, channel surface  does not damage or alter the genomic DNA.

After the DNA samples are passed through the channel, the channel is washed multiple times to flush-out any DNA strands trapped inside the channel. After each wash, gel electrophoresis is carried-out to look for traces of DNA. In Fig. 18(b), the first column is the marker, columns 2, 3, and 4 (5, 6, and 7) show the result after first, second and third washes respectively for run 1 (run 2). Columns 2, 3 and 4 (5, 6, and 7) in Fig. 18(b), show light bands indicating that small amount of DNA are trapped inside the channel and are getting flushed out after each wash. To overcome this issue, adding 2.5% polyvinylpyrrolidone (PVP) in the DNA solution is recommended^31^.

## Conclusions

In this paper, we have presented the design, simulation, and fabrication of low-cost microfluidic channel for biomedical application. Soft lithography technique is used for the channel fabrication. The master was realized on PCB board using tonner transfer technique followed by ferric chloride etching. Issues involved in master fabrication and possible solutions are discussed in detail. PDMS and glass are used as channel and substrate material respectively.

To study the behavior of the channel, simulations were carried-out using COMSOL Multiphysics 5.2a. All the material properties were taken from the COMSOL Multiphysics material library. Microfluidic channel was interfaced with the syringe pump to observe the flow. Also, genomic DNA was passed through the channel to check its biocompatibility and it is found that the channel is suitable enough to handle DNA without causing any damage to it.
